# A −97.2 dB PSR 3.18 ppm/°C Bandgap Voltage Reference with Curvature Compensation

**DOI:** 10.3390/mi17101145

**Published:** 2026-09-30

**Authors:** Hao Wu, Guanghui Shi, Siyan Zhou

**Affiliations:** 1 School of Computer Science, Xi’an Shiyou University, Xi’an 710065, China; miracleworkshop@163.com; 2 Faculty of Integrated Circuit, Xidian University, Xi’an 710000, China; zhou_sy01@163.com

**Keywords:** bandgap reference, high PSR, curvature compensation

## Abstract

Voltage reference has always been an essential building block in integrated circuits and microsystems, such as communication systems and RF modules, as well as wearable and portable devices. This paper proposes a high-power-supply-rejection (PSR) bandgap reference with curvature compensation in the 180 nm BCD process. A common-mode feedback (CMFB) module is integrated into the classical Brokaw bandgap reference to suppress voltage fluctuations at the amplifier input caused by power supply variations. A high-order curvature compensation circuit is used to achieve a low temperature coefficient (TC) by compensating for the nonlinearity of the base-emitter voltage (V_BE_). Simulation results indicate that the circuit realizes a maximum TC of 3.2 ppm/°C from −40 °C to 125 °C with an output voltage of 1.2 V, and an average PSR of −97.2 dB at 100 Hz.

## 1. Introduction

Bandgap reference (BGR) circuits are key components in most integrated microsystems that contain analog or mixed-signal circuits, providing stable and precise voltage references [1,2,3,4]. For example, the signal-processing accuracy of high-performance analog systems such as sensor circuits heavily relies on stable and accurate voltage references. In recent years, the rapid growth of wearable devices, portable devices, as well as medical implants has increased the requirements for signal processing accuracy of microsystems; achieving a bandgap reference with both high PSR and low TC has become a challenge [5,6,7,8].

Conventional BGR designs encounter challenges in achieving a low TC. First-order temperature compensation, commonly used in traditional designs like the Brokaw bandgap reference, is insufficient to meet the stringent demands of modern applications due to the nonlinear temperature dependence of the base-emitter voltage (V_BE_). To address this limitation, an increasing number of designs incorporate high-order curvature compensation to effectively reduce TC [9,10,11,12].

While numerous studies have proposed high-PSR bandgap reference circuits using techniques such as cascode structures and pre-regulators [13,14,15,16,17,18,19,20], theoretical analyses of PSR-enhancement methods are comparatively few. Moreover, existing approaches often lead to increased circuit complexity and large area consumption.

This paper proposes a novel bandgap reference design that achieves both high PSR and low TC by integrating a CMFB module and employing high-order curvature compensation. The CMFB module stabilizes the input voltages of the amplifier against power-supply fluctuations. The CMFB module only requires a single-stage differential amplification chain. By mapping common-mode variations of critical nodes directly to the top current generator, the PSR is improved without compromising the voltage headroom. Simultaneously, a high-order curvature compensation is introduced to mitigate the nonlinearity of V_BE_, resulting in a low TC.

The paper is organized as follows: Section 2 introduces the proposed circuit and provides theoretical analyses of PSR and TC optimization. Section 3 presents the simulation results. The conclusions are included in Section 4.

## 2. Circuit Implementation

**Figure 1:**
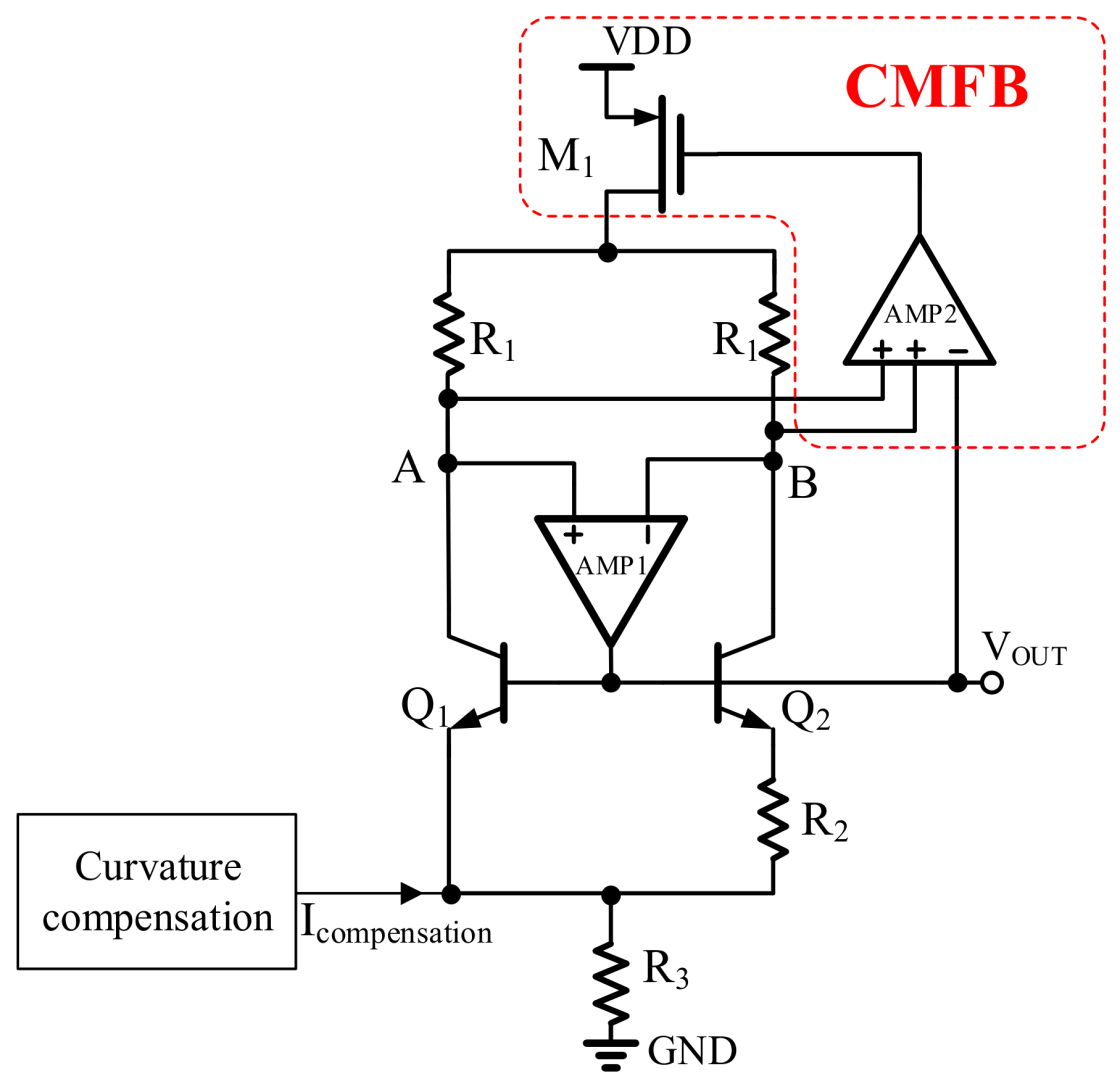
Proposed bandgap reference structure.

**Figure 2:**
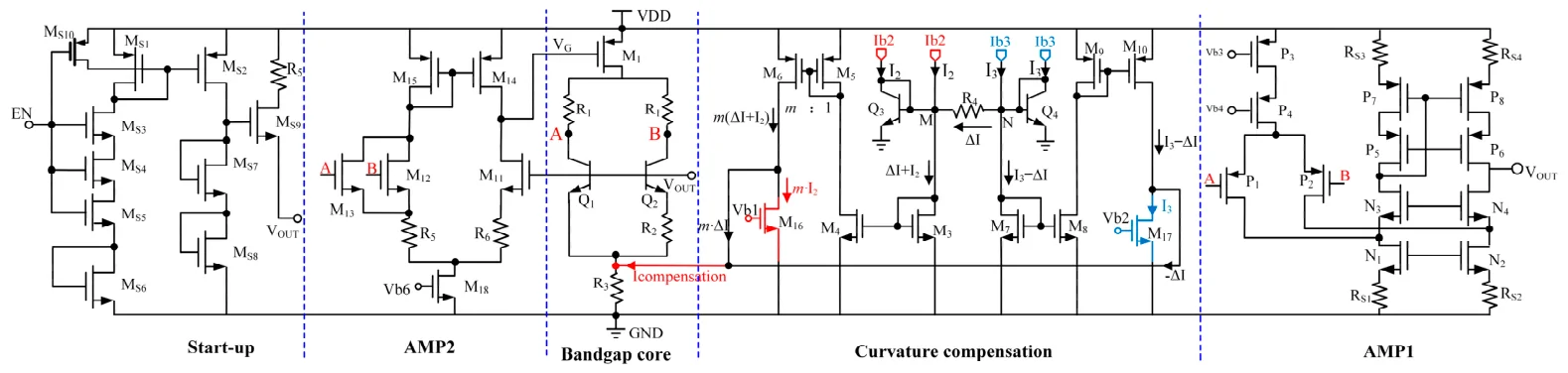
Detailed circuit schematic of the proposed bandgap.

The structure of the proposed bandgap reference is shown in Figure 1, which contains a CMFB module, a curvature compensation module, and a bandgap core circuit. The specific circuit of the proposed structure is shown in Figure 2 in detail.

### 2.1. High Power Supply Rejection Analysis and Design

**Figure 3:**
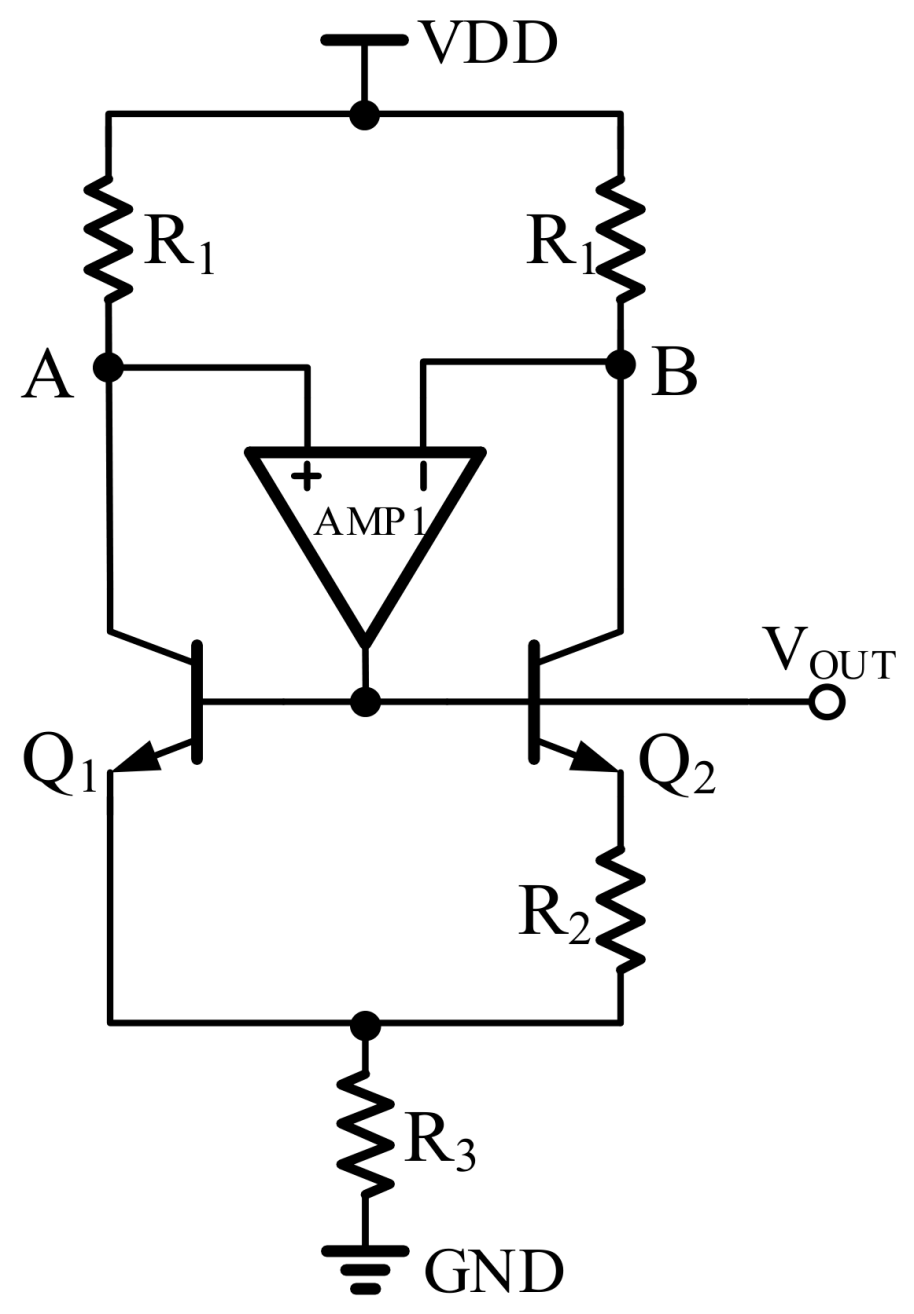
Classical Brokaw bandgap structure.

As shown in Figure 3, the output voltage of the classical Brokaw bandgap is affected by the PSR of AMP1 and voltage variations at nodes A and B. Variations of V_A_ and V_B_ are affected by the supply voltage and the output reference voltage (V_OUT_).

**Figure 4:**
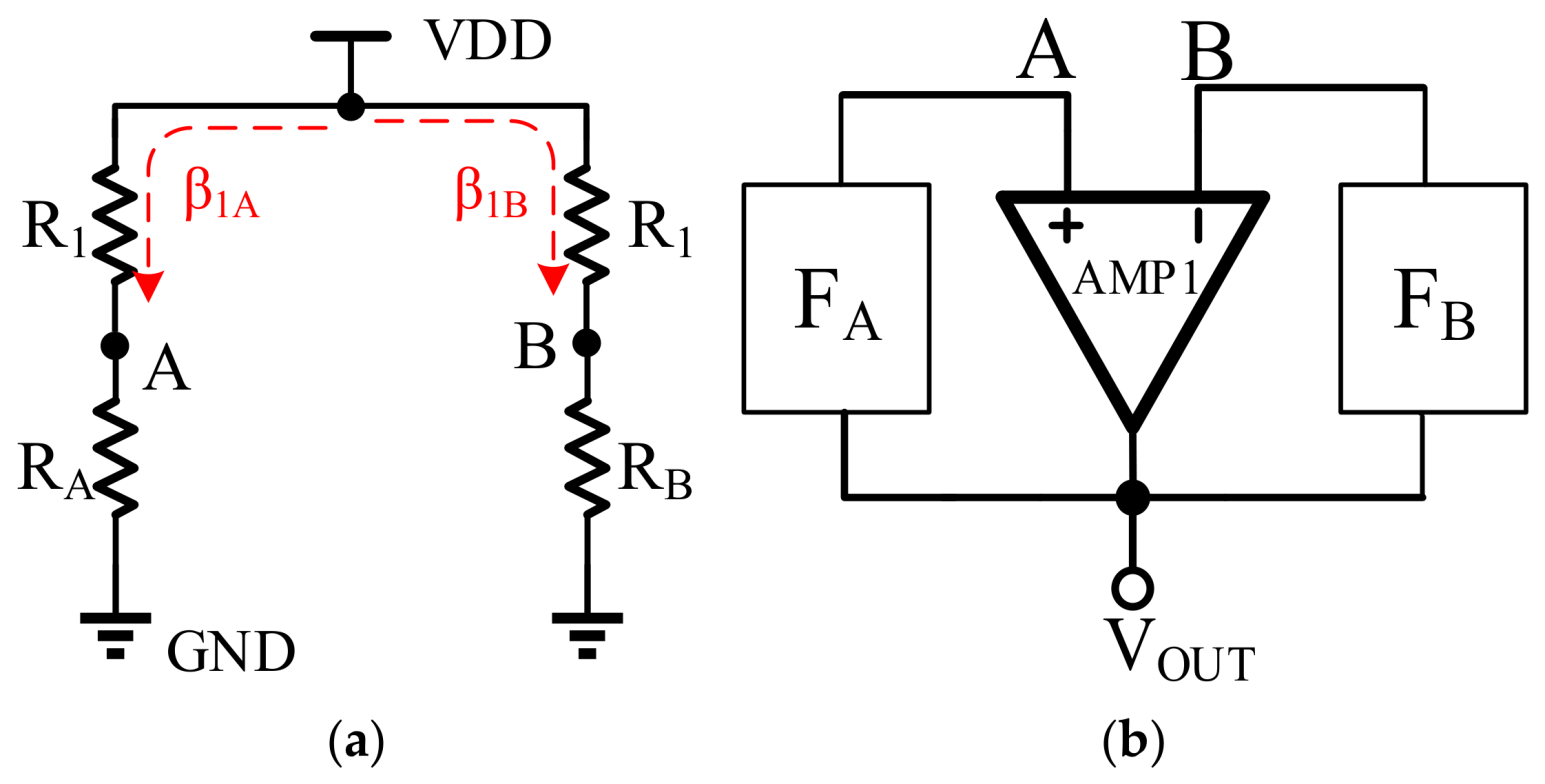
(**a**) Voltage divider; (**b**) Feedback network of the Brokaw BGR.

VDD affects V_A_ and V_B_ through the voltage divider shown in Figure 4a, where β_1A_ is the gain of VDD to V_A_, β_1B_ is the gain of VDD to V_B_; R_A_ denotes the effective impedance from V_A_ to GND, and R_B_ denotes the effective impedance from V_B_ to GND.

(1) $$\beta_{1A}=\frac{R_{A}}{R_{1}+R_{A}}$$

(2) $$\beta_{1B}=\frac{R_{B}}{R_{1}+R_{B}}$$

V_OUT_ affects V_A_ and V_B_ through a feedback network shown in Figure 4b, where F_A_ is a common-emitter (CE) circuit composed of Q_1_, R_1_, and R_3_ with a gain of β_2A_; F_B_ is a CE circuit composed of Q_2_, R_1_, R_2_, and R_3_ with a gain of β_2B_.

(3) $$\beta_{2A}=-\frac{R_{1}}{{2R}_{3}}$$

(4) $$\beta_{2B}=-\frac{R_{1}}{R_{2}{+2R}_{3}}$$

(5) $${\Delta V}_{\mathrm{OUT}}=A_{d}{(\Delta V}_{d,\mathrm{VDD}}+{\Delta V}_{d,\mathrm{OUT}})+A_{\mathrm{cm}}{(\Delta V}_{\mathrm{cm},\mathrm{VDD}}+{\Delta V}_{\mathrm{cm},\mathrm{OUT}})+{\mathrm{PSR}}_{\text{AMP1}}\cdot {\Delta V}_{\mathrm{DD}}$$

The final output voltage variation is shown in Equation (5), where ΔV_VDD_ represents variations in the power supply, ΔV_OUT_ represents the variation of the output, A_d_ represents the differential-mode gain, A_cm_ represents the common-mode gain, and PSR_AMP1_ represents the PSR of AMP1. ΔV_d,VDD_ denotes the difference between V_A_ and V_B_ due to VDD variation, ΔV_d,OUT_ denotes the difference between V_A_ and V_B_ due to V_OUT_ variation, ΔV_cm,VDD_ denotes the common-mode variation between V_A_ and V_B_ due to VDD variation, and ΔV_cm,OUT_ denotes the common-mode variation between V_A_ and V_B_ due to V_OUT_ variation, which can be expressed by

(6) $${\Delta V}_{d,\mathrm{VDD}}=\left(\beta_{1A}-\beta_{1B}\right)\times {\Delta V}_{\mathrm{DD}}$$

(7) $${\Delta V}_{d,\mathrm{OUT}}=\left(\beta_{2A}-\beta_{2B}\right)\times {\Delta V}_{\mathrm{OUT}}$$

(8) $${\Delta V}_{\mathrm{cm},\mathrm{VDD}}={\Delta V}_{\mathrm{DD}}\times \left(\beta_{1A}+\beta_{1B}\right)/2$$

(9) $${\Delta V}_{\mathrm{cm},\mathrm{OUT}}={\Delta V}_{\mathrm{OUT}}\times \left(\beta_{2A}+\beta_{2B}\right)/2$$

For simplicity, use Δβ_n_ to represent (β_nA_ − β_nB_); use ${\bar{\beta }}_{n}$ to represent (β_nA_ + β_nB_)/2. Take Equations (6)~(9) into Equation (5); the ratio of ΔV_OUT_ to ΔV_DD_ can be expressed as

(10) $$\begin{array}{cc} \frac{{\Delta V}_{\mathrm{OUT}}}{{\Delta V}_{\mathrm{DD}}} & =\;A_{d}\;\times \;({\Delta \beta }_{1}\;+{\Delta \beta }_{2}\cdot \frac{{\Delta V}_{\mathrm{OUT}}}{{\Delta V}_{\mathrm{DD}}})+A_{\mathrm{cm}}({\bar{\beta }}_{1}+{\bar{\beta }}_{2}\cdot \frac{{\Delta V}_{\mathrm{OUT}}}{{\Delta V}_{\mathrm{DD}}})+{\mathrm{PSR}}_{\text{AMP1}}\\ & =\frac{A_{d}\;\times \;{\Delta \beta }_{1}+A_{\mathrm{cm}}\;\times \;{\bar{\beta }}_{1}\;{+\mathrm{PSR}}_{\text{AMP1}}}{1-A_{d}\;\times \;{\Delta \beta }_{2}-A_{\mathrm{cm}}\;\times \;{\bar{\beta }}_{2}} \end{array}$$

PSR of the classical Brokaw bandgap reference can be shown as

(11) $$\begin{array}{cc} \mathrm{PSR} & =20\;\mathrm{lg}(\frac{{\Delta V}_{\mathrm{OUT}}}{{\Delta V}_{\mathrm{DD}}})=20\;\mathrm{lg}(\frac{A_{d}\;\times \;{\Delta \beta }_{1}+A_{\mathrm{cm}}\;\times \;{\bar{\beta }}_{1}+{\mathrm{PSR}}_{\text{AMP1}}}{1-A_{d}\;\times \;{\Delta \beta }_{2}-A_{\mathrm{cm}}\;\times \;{\bar{\beta }}_{2}})\\ & =20\;\mathrm{lg}(\frac{{\Delta \beta }_{1}+\frac{{\bar{\beta }}_{1}}{{\mathrm{CMRR}}_{\text{AMP1}}}+\frac{{\mathrm{PSR}}_{\text{AMP1}}}{A_{d}}}{\frac{1}{A_{d}}-{\Delta \beta }_{2}-\frac{{\bar{\beta }}_{2}}{{\mathrm{CMRR}}_{\text{AMP1}}}}) \end{array}$$

where CMRR_AMP1_ is the common-mode rejection ratio, defined as the ratio of differential-mode gain A_d_ to common-mode gain A_cm_.

To achieve a better PSR, the parameters $\frac{{\bar{\beta }}_{1}}{{\mathrm{CMRR}}_{\text{AMP1}}}$ and $\frac{{\mathrm{PSR}}_{\text{AMP1}}}{A_{d}}$ in Equation (11) should ideally approach 0. This necessitates a reference circuit with an infinite CMRR, infinite differential-mode gain (A1), and robust PSR of AMP1.

**Figure 5:**
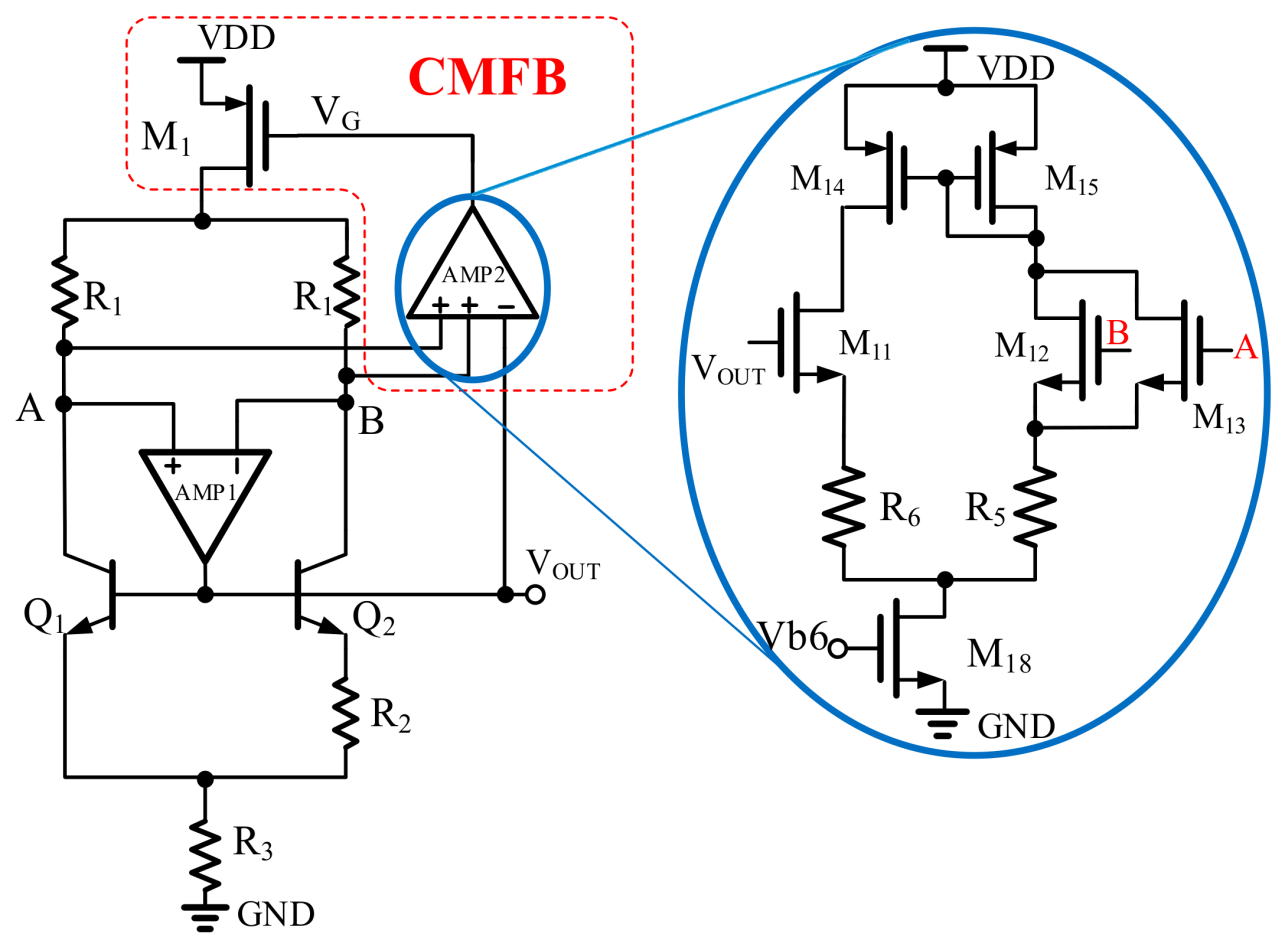
CMFB consists of AMP2 and M1.

To enhance the PSR of the reference circuit in terms of CMRR, two strategies can be employed. Firstly, the internal circuit structure of AMP1 can be optimized to minimize common-mode gain or maximize differential-mode gain. A common method to improve A1 and PSR is the use of cascode configurations. Secondly, supplementary modules can be introduced to stabilize the input voltage, thereby indirectly enhancing common-mode rejection. Therefore, a CMFB module can be incorporated, as shown in Figure 5, where AMP2 and M_1_ form the CMFB module.

The operation of this CMFB can be analyzed as follows. Firstly, an increase in VDD elevates the common-mode voltages of nodes A and B. These voltages serve as in-phase inputs of AMP2, resulting in an amplified output that increases the gate voltage of M_1_. With the introduction of AMP2, the elevated gate voltage of M_1_ reduces the current through M_1_, consequently lowering the common-mode voltages at nodes A and B. Secondly, an increase in V_OUT_ reduces the voltages at nodes A and B via the two common-emitter circuits, F_A_ and F_B_, as depicted in Figure 4b. This decrease in the positive input voltages of AMP2, coupled with an increase in the negative input voltage, leads to a reduction in the gate voltage of M_1_ following differential amplification by AMP2. As a result, the currents through the branches containing Q_1_ and Q_2_ increase, causing the voltages at nodes A and B to rise.

The CMFB module in this work only requires a single-stage differential amplification chain, which does not damage the main pole architecture of the main loop. By mapping the common-mode variations of core nodes directly to the top current generator, strict supply-voltage overhead is avoided.

The proposed structure includes a Brokaw feedback loop which contains AMP1, and a CMFB regulation loop which contains AMP2. The loops must be designed properly to avoid stability issues. The main purpose of the CMFB module is to eliminate power fluctuations at low frequency; therefore, AMP2 is a low-bandwidth, high-DC-gain architecture to make the CMFB loop act as a slow loop. The main Brokaw loop acts as a fast loop; therefore, the bandwidth of AMP1 must be sufficiently larger than that of AMP2. Miller compensation capacitor is utilized to limit the frequency of the main pole of AMP2. As a result, complete decoupling of the main loop and common-mode loop can be achieved in the frequency domain.

### 2.2. Curvature Compensation

High-order curvature compensation is employed to compensate for the nonlinearity of V_BE_, as described in Equation (12) [21]. To eliminate this nonlinearity, a positive term $V_{T}\ln\frac{T}{T_{0}}$ should be introduced through curvature compensation [22].

The expression for V_BE_(T) of a BJT is presented in Equation (12), where V_g0_ is the silicon bandgap voltage at 0 K [23], η is a process-dependent parameter independent of temperature [24], and α is a parameter determined by the temperature coefficient of the bias current flowing through the BJT. Specifically, α equals 1 when the bias current exhibits a positive TC and 0 when the bias current has a zero TC [25].

(12) $$V_{\mathrm{BE}}{(T)=V}_{g0}-{(V}_{g0}-V_{\text{BE0}})\frac{T}{T_{0}}-(\eta -{\alpha )V}_{T}\ln\frac{T}{T_{0}}$$

(13) $$V_{\mathrm{OUT}}=2\frac{R_{3}}{R_{2}}V_{T}{\mathrm{lnN}+V}_{\text{BE1}}$$

**Figure 6:**
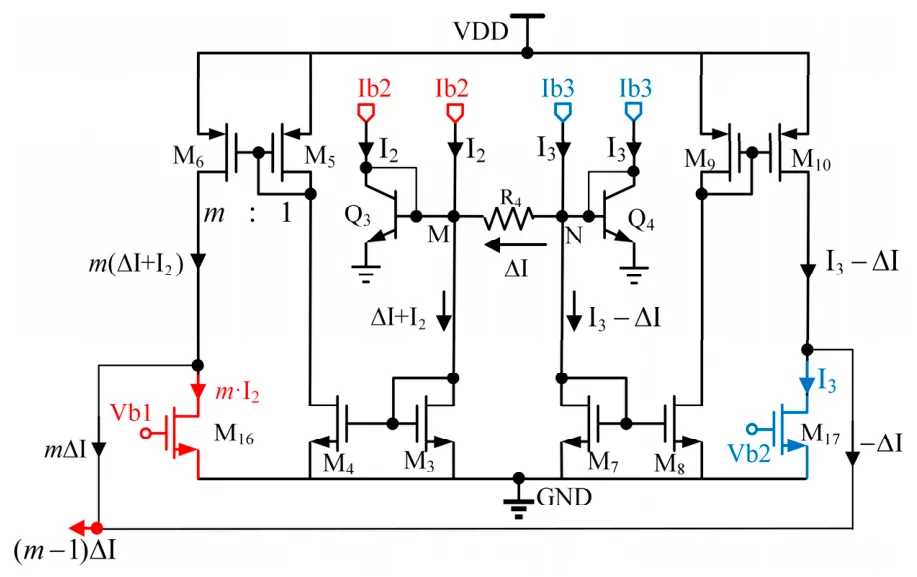
Curvature compensation circuit.

The proposed curvature compensation circuit is illustrated in Figure 6. The current I_2_ flowing through Q_3_ is a constant current, whereas the bias current I_3_ flowing through Q_4_ is proportional to the absolute temperature (PTAT) current. The V_BE_ expressions of Q_3_ and Q_4_ are provided in Equations (14) and (15), respectively. By taking the difference between the V_BE_ of Q_3_ and Q_4_, the required term is obtained, as shown in Equation (16). This voltage is converted into a current ΔI via resistor R_4_ as expressed in Equation (17).

As depicted in Figure 6, the current flowing through M_3_ is ∆I + I_2_. This current is mirrored and scaled to *m*(∆I + I_2_) by the current mirror comprising M3–M6, where M_5_ and M_6_ are designed with a ratio of 1: *m*. Similarly, the current mirror M_7_–M_10_ replicates the current I_3_ − ∆I flowing through M_7_. The total compensation current is composed of the sum of two components, *m*ΔI and −ΔI, as expressed in Equation (18).

(14) $$V_{{\mathrm{BE},Q}_{4}}{(T)=V}_{g0}-{(V}_{g0}-V_{\text{BE0}})\frac{T}{T_{0}}-(\eta -{1)V}_{T}\ln\frac{T}{T_{0}}$$

(15) $$V_{{\mathrm{BE},Q}_{3}}{(T)=V}_{g0}-{(V}_{g0}-V_{\text{BE0}})\frac{T}{T_{0}}-{\eta V}_{T}\ln\frac{T}{T_{0}}$$

(16) $${\Delta V}_{{\mathrm{BE},Q}_{4}-Q_{3}}{(T)=V}_{T}\ln\frac{T}{T_{0}}$$

(17) $$\Delta I=\frac{V_{T}\ln T/T_{0}}{R_{4}}$$

(18) $$I_{\mathrm{compensation}}=(m-1)\Delta I=\frac{m-1}{R_{4}}V_{T}\ln T/T_{0}$$

With the incorporation of the curvature compensation module, the output voltage of the bandgap reference is adjusted from Equation (13) to Equation (19).

(19) $$V_{\mathrm{OUT}}=2\frac{R_{3}}{R_{2}}V_{T}{\mathrm{lnN}+V}_{\text{BE1}}+\frac{R_{3}}{R_{4}}(m-{1)V}_{T}\ln\frac{T}{T_{0}}$$

By substituting the temperature-dependent expression of V_BE_ given in Equation (12) into Equation (19), we obtain Equation (20).

(20) $$V_{\mathrm{OUT}}=2\frac{R_{3}}{R_{2}}\frac{\mathrm{kT}}{q}\mathrm{lnN}+[V_{g0}-{(V}_{g0}-V_{\text{BE0}})\frac{T}{T_{0}}-(\eta -\alpha )\frac{\mathrm{kT}}{q}\ln\frac{T}{T_{0}}]+\frac{R_{3}}{R_{4}}(m-1)\frac{\mathrm{kT}}{q}\ln\frac{T}{T_{0}}$$

Taking the derivative of Equation (20) with respect to temperature yields Equation (21).

(21) $$\frac{{\mathrm{dV}}_{\mathrm{OUT}}}{\mathrm{dT}}=2\frac{R_{3}}{R_{2}}\frac{k}{q}\mathrm{lnN}-\frac{V_{g0}-V_{\text{BE0}}}{T_{0}}+[\frac{R_{3}}{R_{4}}(m-1)-(\eta -\alpha )]\frac{k}{q}(\ln\frac{T}{T_{0}}+1)$$

Since only the last term of Equation (21) contains the temperature variable, setting Equation (21) to zero yields only one solution. Therefore, this compensation approach leads to a temperature curve with a single zero-slope point under ideal conditions. The temperature value corresponding to zero slope in the output curves before and after curvature compensation is different, which results from the different coefficients of the temperature variable in the last term of Equation (21).

With properly designed values of *m* and the ratio of resistors, the influence of high-order nonlinear temperature terms on the reference voltage can be effectively weakened. To minimize layout-induced mismatch non-idealities, *m* and the ratio of resistors should be restricted to integers. The objective is to perfectly neutralize the non-linear component of V_BE_ at the central temperature node, achieving the minimum temperature coefficient. Therefore, the zero-slope point should be located around the middle of the concerned temperature span.

### 2.3. Bias Circuit

**Figure 7:**
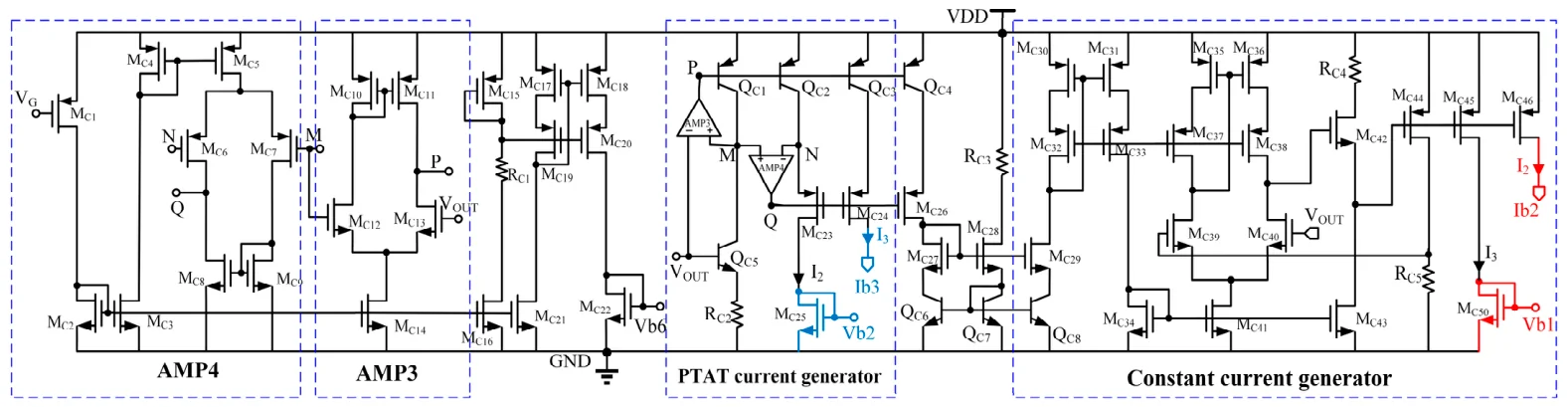
Bias generator for curvature compensation.

Figure 7 illustrates the bias-generation circuit employed in the curvature compensation module. In the PTAT current generator, the bandgap reference output voltage is connected to the base of transistor Q_C5_. Since the base–emitter voltage V_BE_ of Q_C5_ has a negative temperature coefficient and V_OUT_ is temperature independent, the emitter voltage of Q_C5_, which corresponds to the voltage across resistor R_C2_, shows a positive temperature coefficient. The current flowing through R_C2_ therefore has a positive temperature coefficient. This current is mirrored by transistors Q_C1_~Q_C3_ to generate a PTAT current I_3_. The bias voltage Vb2 is applied to the gate of M_17_ in Figure 6. AMP3 and AMP4 are employed as clamping amplifiers. In AMP4, the gate voltage V_G_ of M_C1_ is driven by the output of the CMFB amplifier AMP2, which also controls the gate of M_1_. Under normal operation of the CMFB module, M_1_ in Figure 5 and M_C1_ in Figure 7 are simultaneously biased on.

In the constant-current generator, the amplifier formed by M_C35_~M_C44_, R_C4_, and R_C5_ provides strong negative feedback, forcing the inverting input at the gate of M_C39_ to closely track the non-inverting input voltage V_OUT_. Therefore, under temperature variations, the voltage across R_C5_ remains approximately constant. The resulting nearly constant current is then mirrored by the current mirror formed by M_C45_~M_C46_ to generate I_2_. This current is used to generate the bias voltage Vb1, which is applied to the gate of M_16_ in Figure 6, and M_16_ mirrors the PTAT current with a scaling factor of *m*.

## 3. Post-Layout Simulation Results

**Figure 8:**
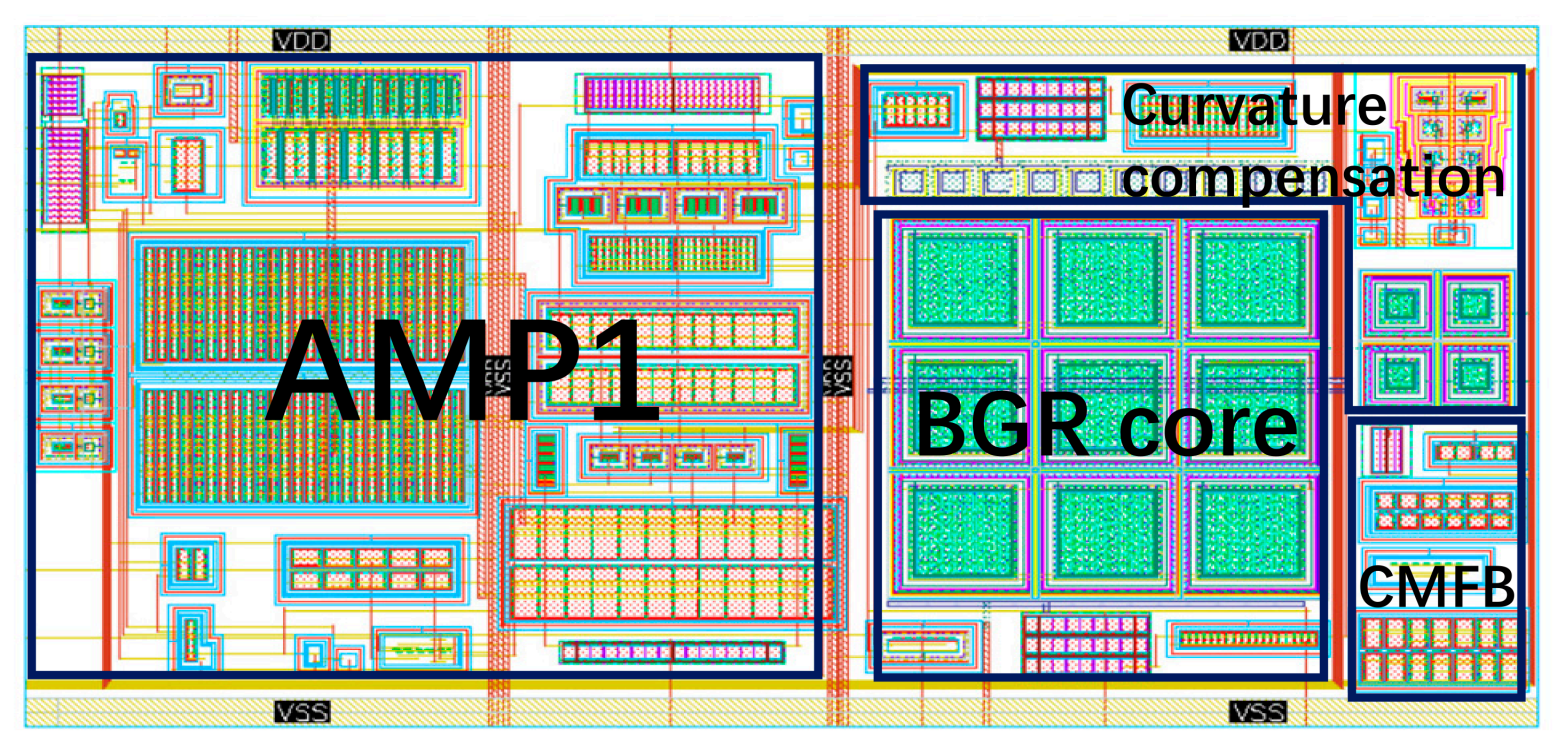
The layout of the proposed BGR.

The proposed BGR circuit is implemented using the 0.18 um process and occupies an active area of 0.118 mm^2^, which is depicted in Figure 8.

**Figure 9:**
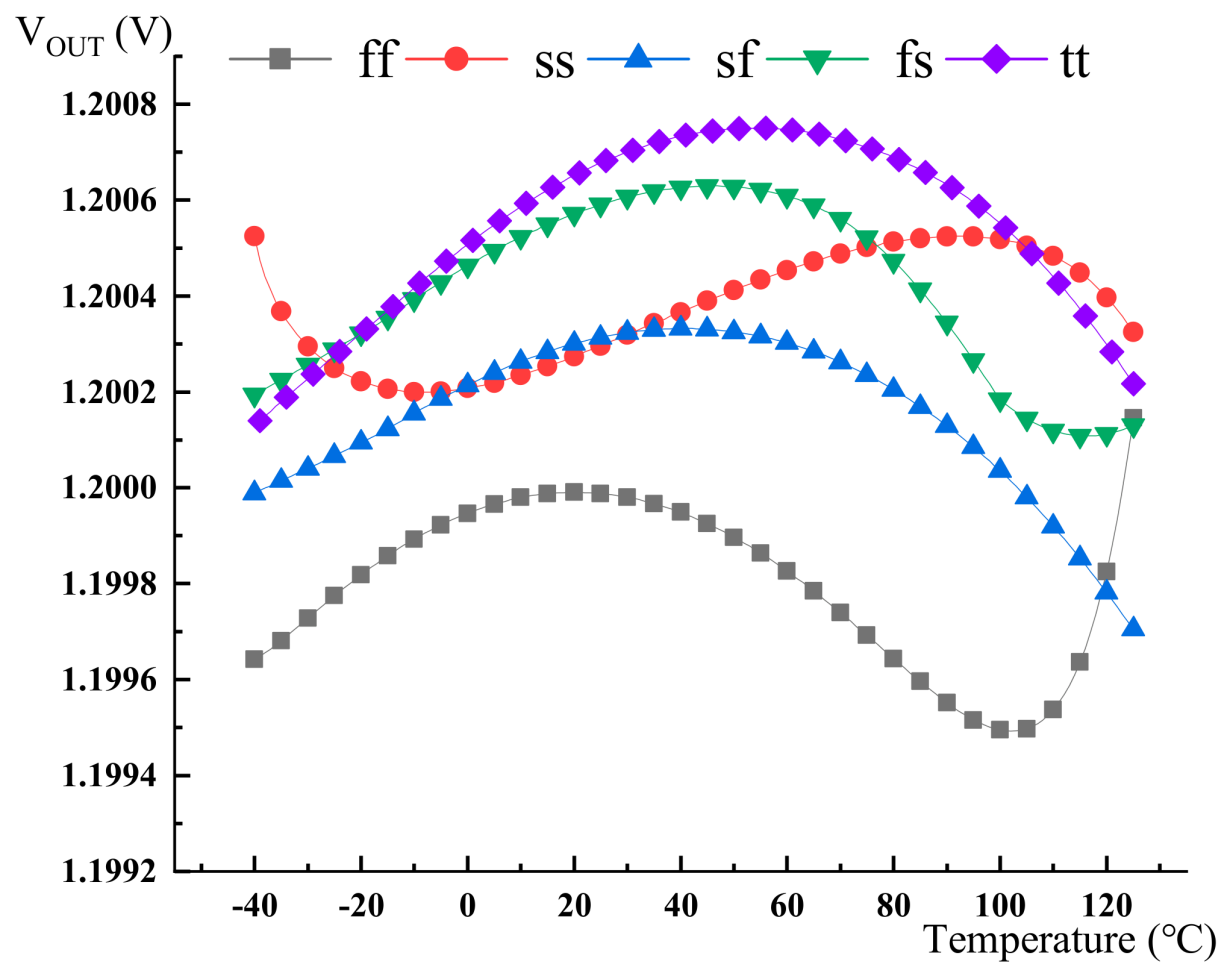
The V_OUT_-T curve at different process corners.

The proposed design shows load regulation of 2.14~2.6 mV/mA across process corners, and 2.28 mV/mA at the tt corner. Figure 9 shows the post-simulation result of output voltage from −40 to 125 °C at process corners. The tt, ff, ss, sf, and fs corners present TC of 3.07 ppm/°C, 2.63 ppm/°C, 1.66 ppm/°C, 2.62 ppm/°C, and 3.18 ppm/°C, respectively. The appearance of multiple zero-slope points under the ff and ss process corners is not an intended design feature. Instead, they result from process variations. Actually, the single zero-slope expression is derived with an ideal device model, which assumes the temperature has no effect on the gain of amplifiers, BJT ratios, and resistance ratios, which will all appear in the layout simulation. Moreover, I_2_ and I_3_ are not ideal constant current or PATA current, and they are actually generated on the basis of V_OUT_, which is temperature-dependent itself. In other words, the effect of temperature on parameter α can emerge under extreme processes. However, the TC is not degraded under extreme processes; therefore, practical application is not affected.

**Figure 10:**
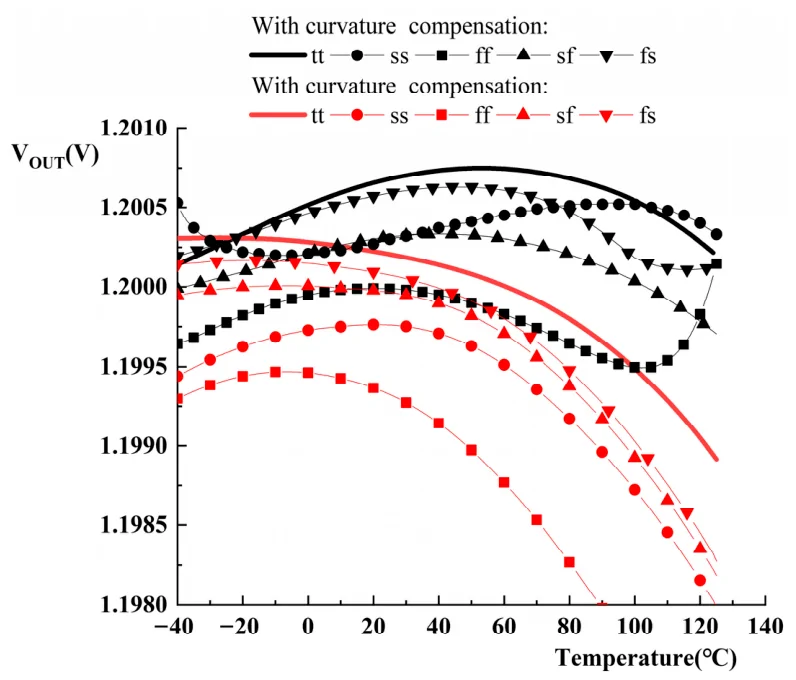
Comparison between V_OUT_ with and without curvature compensation.

Figure 10 compares the output V_OUT_-T curves from −40 °C to 125 °C with and without curvature compensation at process corners. At tt corner, the TC is 3.07 ppm/°C with curvature compensation and 7.57 ppm/°C without curvature compensation, and the zero-slope temperature changes when curvature compensation is applied. This originates from the additional coefficient in the last temperature-dependent term of Equation (21); the value of T making dV_OUT_/dT equal to 0 is actually increased when the compensation term is introduced. Furthermore, because the original nonlinear temperature component in Equation (20) is compensated, the voltage shows a smaller variation over temperature.

**Figure 11:**
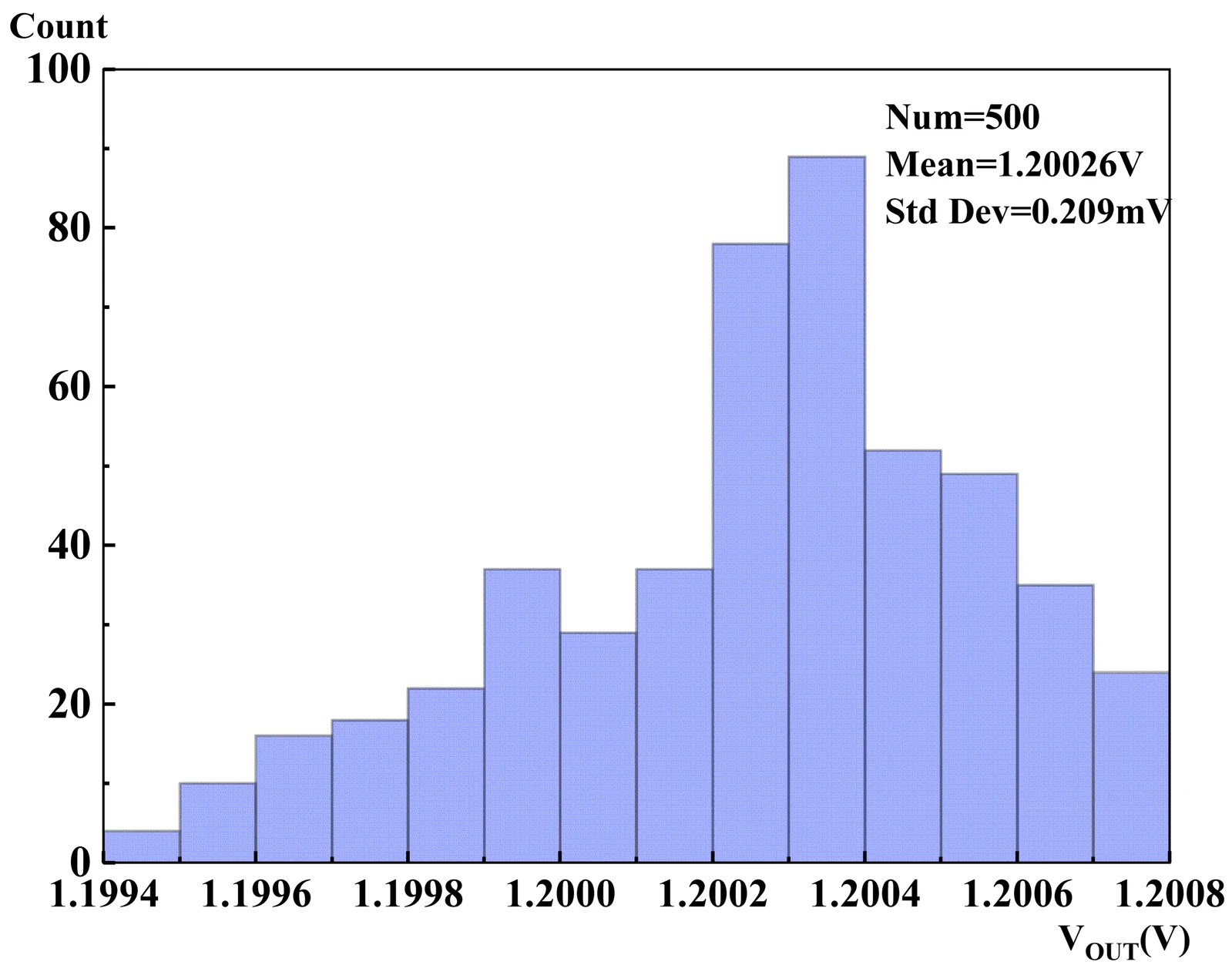
500-point Monte Carlo simulation results of V_OUT_.

Figure 11 is the output voltage distribution obtained from 500-point Monte Carlo analyses, with an average output voltage of 1.20026 V and a standard deviation of 0.209 mV.

**Figure 12:**
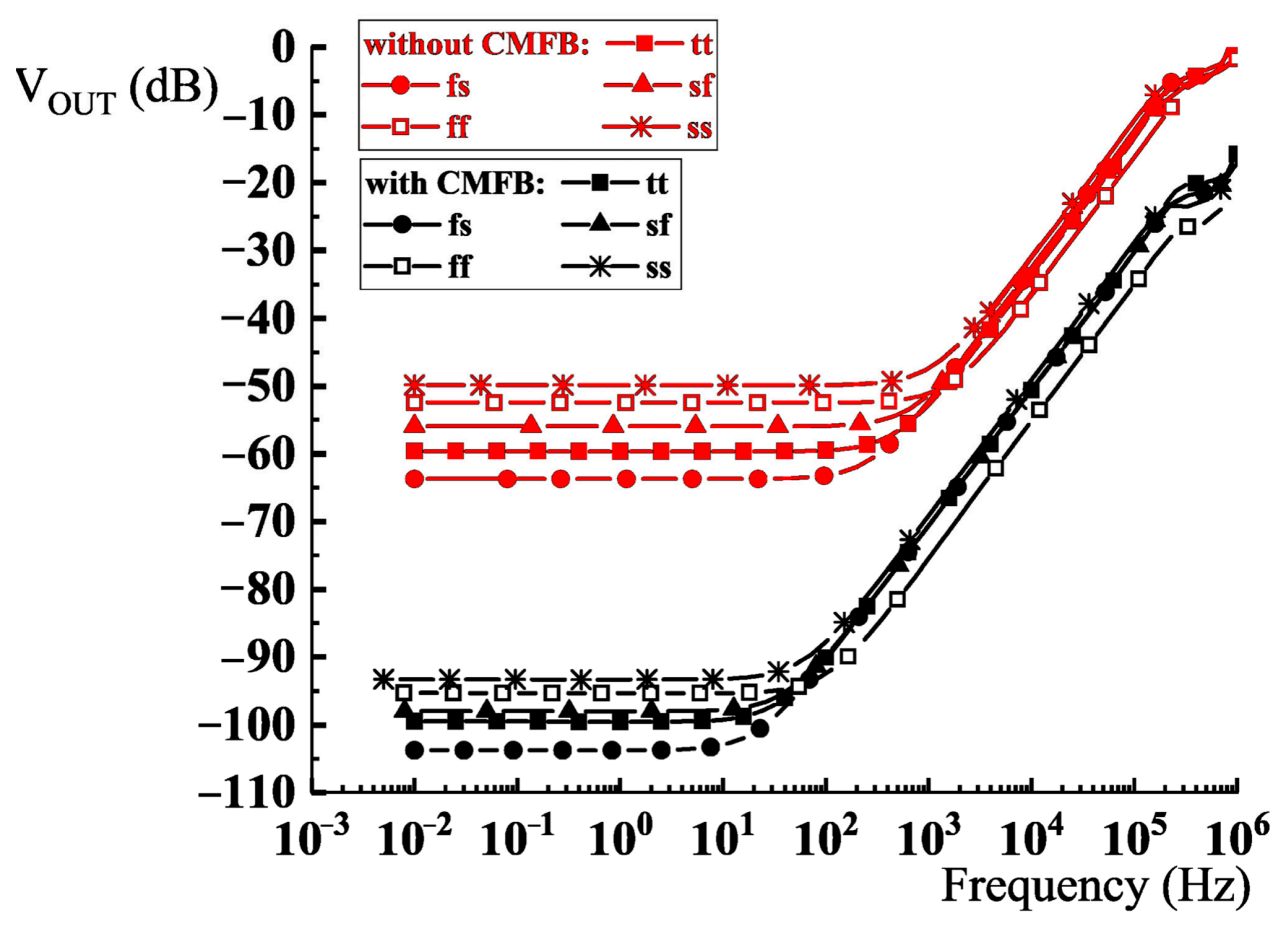
The PSR performance with and without CMFB across corners.

Figure 12 presents a comparison between the PSR performance with and without CMFB. At the TT corner, the low-frequency PSR is −59 dB without CMFB and improves significantly to −96.2 dB with CMFB, which validates the theoretical analysis of PSR and demonstrates that the incorporation of CMFB enhances the PSR performance of the classical Brokaw bandgap. Actually, the PSR roll-off arises when the CMFB loop gain drops with frequency. As discussed earlier, the bandwidth of AMP2 is designed to be very low. As frequency increases, the CMFB loop gain drops and disturbance is no longer suppressed. Miller compensation in AMP2 results in a low frequency of the dominant pole; therefore, the gain of AMP2 A_AMP2_ can be approximated by

(22) $$A_{\text{AMP2}}(s)=\frac{A_{\mathrm{dc},2}}{1+\frac{s}{\omega_{p2}}}$$

where A_dc,2_ is the DC gain of AMP2 and ω_p2_ is the dominant pole of AMP2. The load impedance at M1 drain can be expressed as

(23) $$Z_{\mathrm{AB}}(s)=R_{\mathrm{eq}}\left|\right|\frac{1}{{\mathrm{sC}}_{\mathrm{para}}}=\frac{R_{\mathrm{eq}}}{{1+\mathrm{sR}}_{\mathrm{eq}}C_{\mathrm{para}}}$$

where R_eq_ and C_para_ are the equivalent resistance and parasitic capacitance seen at M1 drain. Assuming g_m1_ is the transconductance of M1, the CMFB loop gain G_CMFB_(s) can be expressed as

(24) $$G_{\mathrm{CMFB}}{(s)=A}_{\text{AMP2}}\left(s\right)\cdot g_{m1}\cdot Z_{\mathrm{AB}}(s)=\frac{A_{\mathrm{dc},2}g_{m1}R_{\mathrm{eq}}}{\left(1+\frac{s}{{1\;/\;R}_{\mathrm{eq}}C_{\mathrm{para}}}\right)\left(1+\frac{s}{\omega_{p2}}\right)}$$

There are two poles in the approximated loop gain. However, the dominant pole of AMP2 is limited to be very low through Miller compensation to form a slow loop; therefore, ω_p2_ is much smaller than $\frac{1}{R_{eq}C_{par}}$ and the PSR corner frequency is limited by the bandwidth of AMP2.

**Figure 13:**
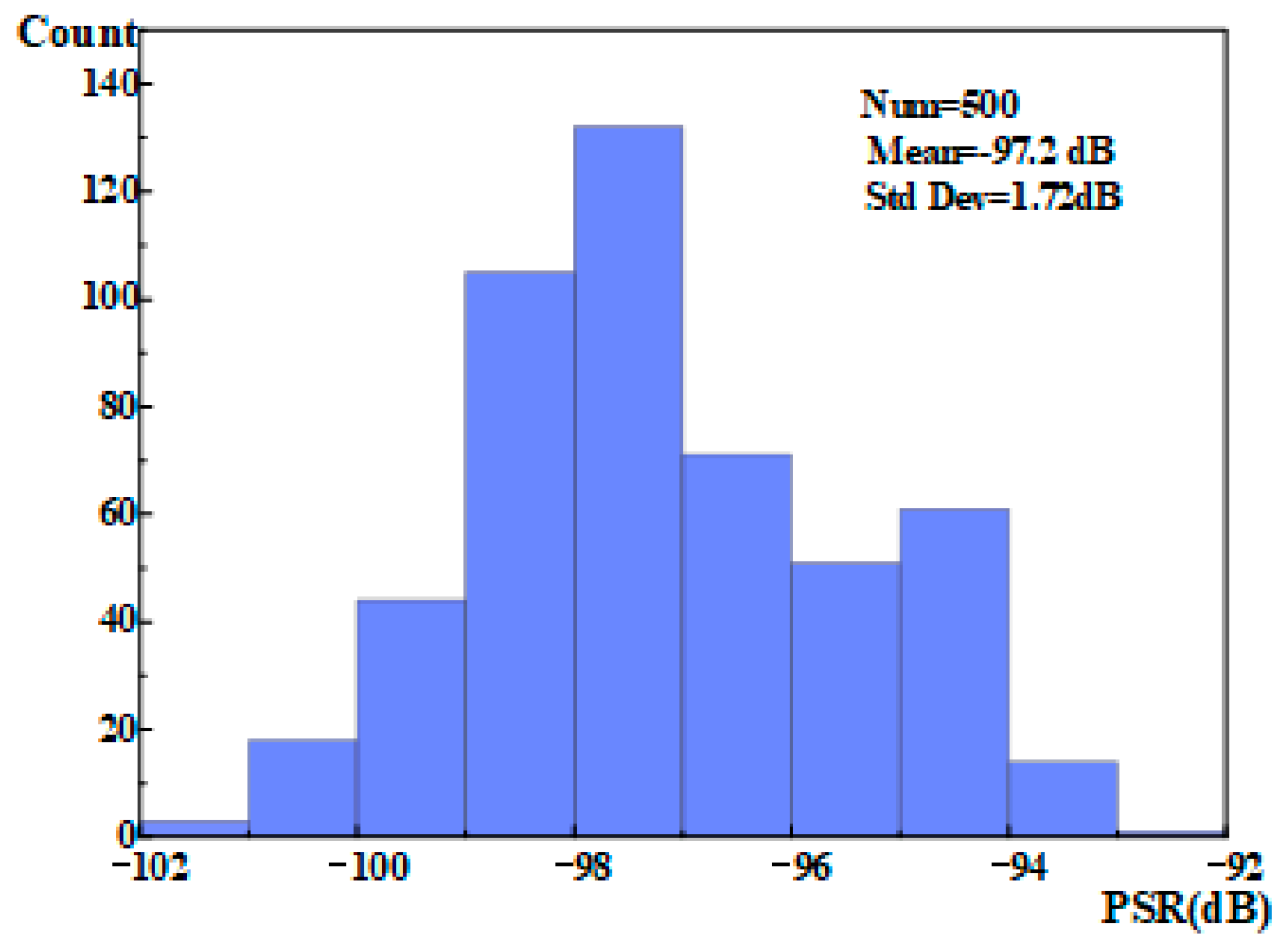
500-point Monte Carlo simulation results of PSR at 100 Hz.

Figure 13 is the PSR distribution at 100 Hz obtained from 500-point Monte Carlo analyses of the proposed BGR circuit, with an average PSR of −97.2 dB and a standard deviation of 1.72 dB.

**Figure 14:**
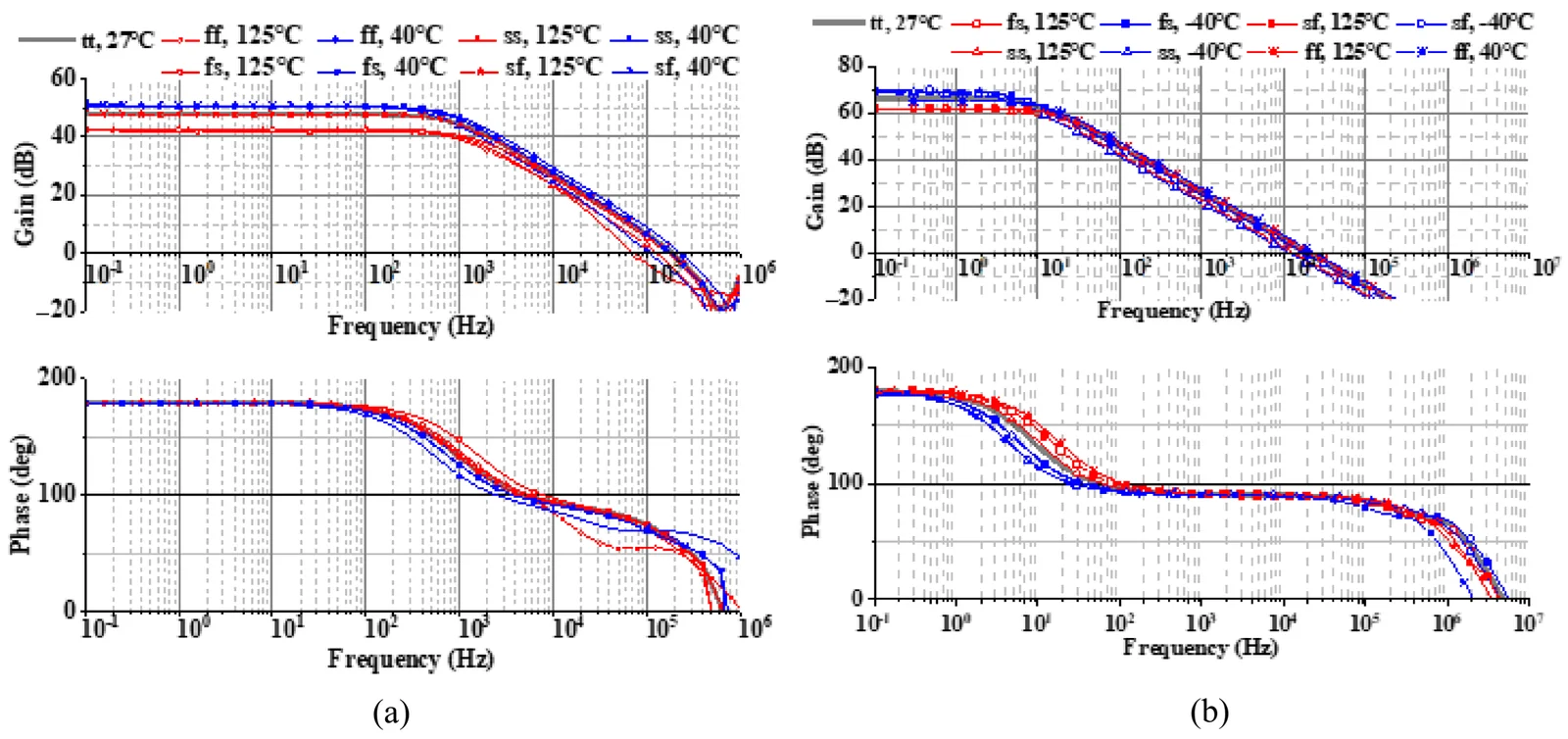
Bode plot for the loop gain: (**a**) Brokaw loop; (**b**) CMFB loop.

The loops must be designed properly to avoid stability risks. The Bode plot for both the main loop and CMFB loop across process corners and temperature extremes is shown in Figure 14. Since Miller compensation is adopted to limit the frequency of the main pole of AMP2, the bandwidth of the CMFB loop is an order of magnitude smaller than that of the main loop, achieving decoupling of the main loop and common-mode loop in the frequency domain.

**Figure 15:**
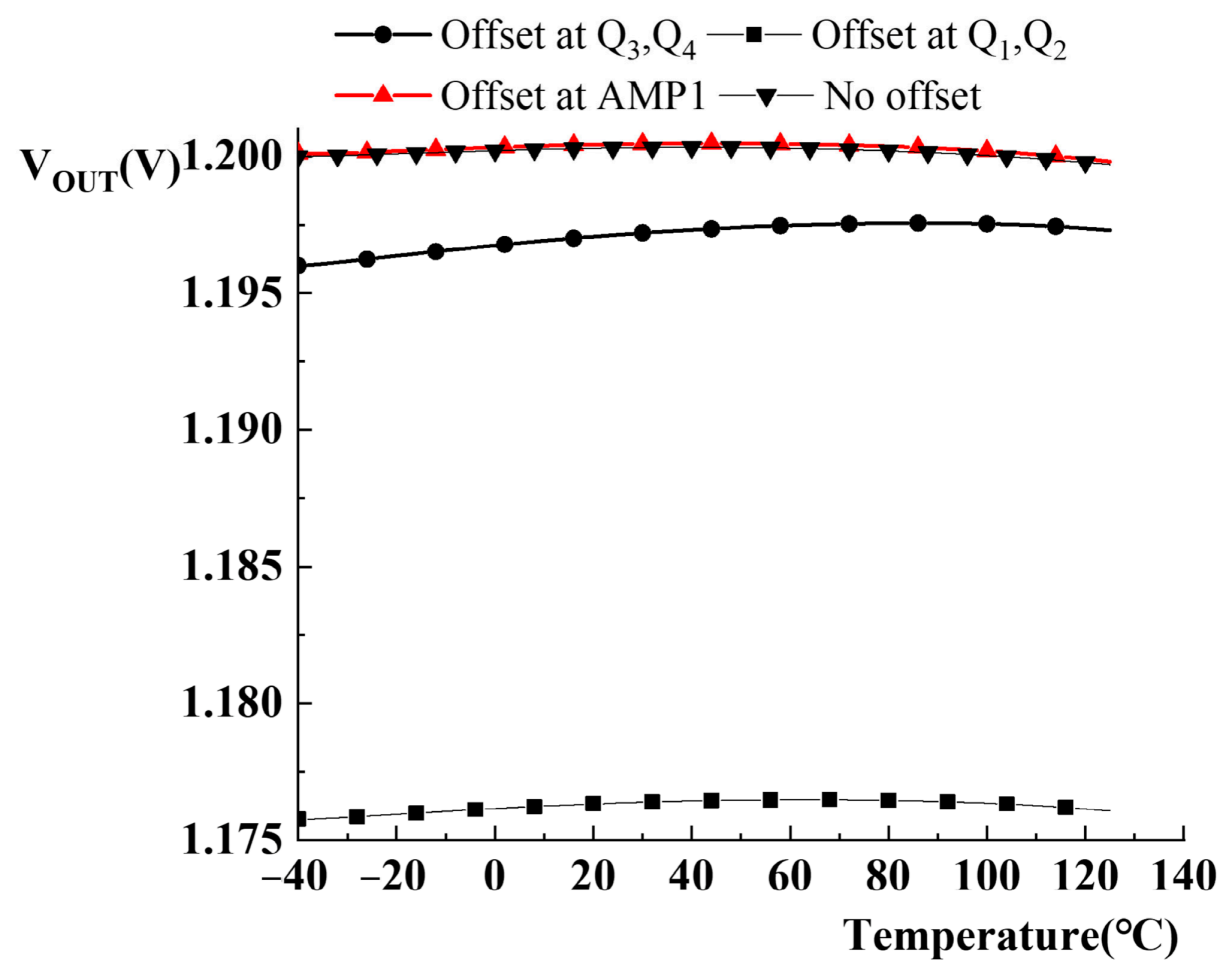
Simulated V_OUT_ with offset voltages of 2 mV.

It is necessary to mention that the results are based on post-layout simulations. Non-idealities could degrade the real-chip performance. For example, at elevated temperatures, leakage current from the current mirrors can become significant, which injects directly into node A/B or the bipolar transistors. The TC curve relies on the ratio through Q_1_/Q_2_ and the compensation current, both of which will be corrupted by the leakage current. Furthermore, the decrease in differential-mode gain and CMRR will degrade PSR. On the other hand, unpredictable packaging stress could induce physical mismatch in internal resistors and transistors, resulting in absolute drift in the output voltage and severe degradation of the temperature coefficient. Figure 15 depicts the effect of a 2 mV offset voltage V_os_ at the input pairs of AMP1 and the BJT pairs (Q1, Q2 and Q3, Q4). Mathematically, V_os_ at the AMP1 input will only add a small DC term to the output voltage through ΔV_be_; therefore, TC is not influenced. V_os_ at Q_1_/Q_2_ pairs causes significant degradation of V_OUT_, because it is magnified to about 20 mV by the amplification factor of the PTAT term V_OUT_ in Equation (13). V_os_ at Q_3_/Q_4_ pairs degrades the TC to 8 ppm/°C because the term for compensation in Equation (20) is disturbed. However, in real chips, V_os_ is actually strongly temperature-dependent, and the TC could be a very complex nonlinear function. In addition, real-world chips suffer from unavoidable overlapping random offsets; therefore, trimming is necessary.

Table 1 summarizes the performance of the proposed BGR in comparison to other reported results. The proposed design achieves both a combination and a balance of high PSR and low TC while maintaining a compact layout area.

## 4. Conclusions

A low TC, high-PSR bandgap reference with curvature compensation is proposed and implemented by the 180 nm BCD process in this paper. The design incorporates high-order curvature compensation to reduce nonlinearity in VBE, and employs a common-mode feedback module to enhance the PSR performance. As a result, the proposed reference achieves a maximum TC of 3.18 ppm/°C over a temperature range from −40 °C to 125 °C across process corners. Furthermore, compared to the conventional Brokaw topology, the PSR at 100 Hz is significantly improved from −59 dB to −96.2 dB with the introduction of the common-mode feedback module. These results demonstrate that the proposed architecture achieves a well-balanced combination of wide temperature stability, high PSR, and compact layout, making it a practical solution for high-precision sensor interface systems such as medical implants, aerospace, and Internet of Things (IoT).

## Figures and Tables

**Table 1:** Performance comparison.

	[9]	[16]	[26]	[27]	[28]	[29]	This Work
Process (μm)	0.18	0.18	0.18	0.11	0.18	0.18	0.18
Vref (V)	1.038	1.245	1.21	1.2	1.65	1.25	1.2
Tem. Range (°C)	−55~125	−40~130	−32~135	−40~125	−40~125	−45~125	−40~125
TC (ppm/°C)	0.2	3.25	0.7~1.16	2.18	2.2~6.2	4.2	1.66~3.18
PSR (dB) @100 Hz	−71	−103	−89	N/A	−68	−100	−97.2
Quiescent current (μA)	49	N/A	N/A	N/A	N/A	N/A	32
Power (μW)	161.7	13.2	N/A	N/A	N/A	127.8	160
Line Regulation (%/V)	N/A	N/A	N/A	N/A	0.18	0.012	0.27
Active area (mm^2^)	0.01	0.56	0.0695	N/A	0.329	1.3	0.118

## Data Availability

The original contributions presented in this study are included in the article. Further inquiries can be directed to the corresponding author.
